# Analysis of Deflection Enhancement Using Epsilon Assembly Microcantilevers Based Sensors

**DOI:** 10.3390/s111009260

**Published:** 2011-09-28

**Authors:** Abdul-Rahim A. Khaled, Kambiz Vafai

**Affiliations:** 1 Thermal Engineering and Desalination Technology Department, King Abdulaziz University, P.O. 80204, Jeddah 21589, Saudi Arabia; E-Mail: akhaled@kau.edu.sa; 2 Mechanical Engineering Department, University of California, Riverside, CA 92521, USA

**Keywords:** microcantilever, assembly, detection, deflection, enhancement

## Abstract

The present work analyzes theoretically and verifies the advantage of utilizing ɛ-microcantilever assemblies in microsensing applications. The deflection profile of these innovative ɛ-assembly microcantilevers is compared with that of the rectangular microcantilever and modified triangular microcantlever. Various force-loading conditions are considered. The theorem of linear elasticity for thin beams is used to obtain the deflections. The obtained defections are validated against an accurate numerical solution utilizing finite element method with maximum deviation less than 10 percent. It is found that the ɛ-assembly produces larger deflections than the rectangular microcantilever under the same base surface stress and same extension length. In addition, the ɛ-microcantilever assembly is found to produce larger deflection than the modified triangular microcantilever. This deflection enhancement is found to increase as the ɛ-assembly’s free length decreases for various types of force loading conditions. Consequently, the ɛ-microcantilever is shown to be superior in microsensing applications as it provides favorable high detection capability with a reduced susceptibility to external noises. Finally, this work paves a way for experimentally testing the ɛ-assembly to show whether detective potential of microsensors can be increased.

## 1. Introduction

The rapid growth of nanotechnology has led to the development of new sensing devices of micrometer size coined as microsensors. These devices can be used to detect, measure, analyze, and economically monitor low concentrations of chemical and biological agents. The monitoring of a specific substance is pivotal in many applications, especially for clinical purposes in order to screen a patient for the presence of a disease at an early stage [1]. Microcantilever-based microsensors have been proven to be very sensitive and accurate [1]. The changes in the physical properties of the microcantilever are considered to indicate or detect changes in the environment surrounding it [2,3]. These changes can for example be measured using electric signals with piezoressitive microcantilevers [2–4]. They can also be gauged by monitoring the tip deflection of the microcantilevers [5–7]. The deflection of the microcantilever was first used for atomic force microscopy [5]. Moreover, the changes in the physical properties of the microcantilever are widely used to indicate the presence or absence of a certain analyte [8–11].

The magnitude of microcantilever deflection is of the order of nanomenters and it is usually measured using optical methods. The performance of the microcantilever as a sensing device is affected by the noise level in the surrounding environment. For example, Fritz *et al*. [12] reported that the microcantilever deflection due to flow disturbances and due to thermal effects could reach 5–10 times that due to analyte sensing. Accordingly, further developments in microcantilever technology are necessary in order to magnify the deflection signal due to the sensing effect so that its signal can be easily distinguished from the noise signal [13–16]. As such, Khaled *et al*. [2] pointed out the necessity of establishing special microcantilevers assemblies for this purpose. Many of these assemblies were patented [14,17]. It should be noted that additional novel methods for magnifying the deflection signal due to analyte sensing were proposed [18–21]. Some of these methods are based on controlling both the geometry of the fluidic cell incubating the microcantilevers and their geometrical distribution. An interesting assembly among the assemblies described in the work of Khaled *et al*. [2] is the ɛ-microcantilever assembly. The deflection due to analyte sensing of ɛ-microcantilever assembly is estimated to be double that of the rectangular microcantilever [2]. As such, this assembly is considered to be highly important for the present work.

In this work, the advantage of utilizing microcantilever assemblies including the ɛ-assembly established by Khaled *et al*. [2] in microsensing applications is explored theoretically. Various force loading conditions that can produce noticeable deflections such as the concentrated force, moment and constant surface stress which can be due to analyte adhesion are considered. The linear elasticity theory for thin beams [22] is used to obtain the deflections. Different deflection indicators are defined and various controlling variables are identified. The performance of different microcantilever assemblies is compared with the performance of rectangular microcantilevers in order to map out conditions that produce magnification of the sensing deflection relative to the noise deflection.

## 2. Theoretical Analysis

### 2.1. Microcantilevers with One Piece (Rectangular Microcantilevers)

**Figure 1.**
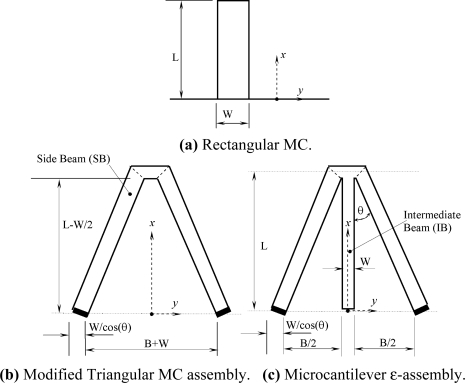
Schematic diagrams and the corresponding coordinate system for microcantlievers (MC) assemblies: **(a)** Rectangular MC; **(b)** the modified Triangular MC assembly; and **(c)** the ɛ-MC assembly.

The geometry of the rectangular microcantilever considered in this section is shown in Figure 1(a). The properties of the rectangular microcantilever can be summarized by specifying the extension length *L*, width *W*, thickness *t*, Young’s modulus *E* and Poisson’s ratio *ν*. When the length of the microcantilever is much larger than its width, Hooks law for small deflections can be used to relate the microcantilever deflections to the effective elastic modulus *Y* and the bending moment *M* [22]. It is given by:

(1) $$\frac{d^{2}z}{dx^{2}}=\frac{M}{\text{YI}}$$

where z is the deflection the microcantilever at any section located at a position *x* from the base surface. *I* is the area moment of inertia of the microcantilever cross-section about its neutral axis. For a rectangular cross-section with its neutral axis coinciding with its centroidal axis, *I* is given by:

(2) $$I=\frac{1}{12}{\mathrm{Wt}}^{3}$$

The boundary conditions for Equation (1) are given by:

(3a,b) $$z\left(x=0\right)={\frac{\mathrm{dz}}{\mathrm{dx}}|}_{x=0}=0$$

For a concentrated force exerted on the rectangular microcantilever tip (*x* = *L*), the solution of Equation (1), denoted by *z_aF_*(*x*), subject to boundary conditions given by Equation 3(a,b) can be expressed as:

(4) $$z_{\mathrm{aF}}\;\left(x\right)=\left(\frac{6{\mathrm{FL}}^{3}}{{\mathrm{EWt}}^{3}}\right)\left[{\left(\frac{x}{L}\right)}^{2}-\frac{1}{3}{\left(\frac{x}{L}\right)}^{3}\right]$$

The above result is based on a realistic linearly increasing bending moment from the base prescribed by:

(5) $$M=\mathrm{FL}\left(1-\frac{x}{L}\right)$$

For thin cross-sections, the surface stress, σ, can be calculated from the following equation:

(6) $$\sigma =\frac{M}{I}\left(\frac{t}{2}\right)$$

The surface stress at *x =* 0 (base surface) denoted by *σ_aFo_* is equal to:

(7) $$\sigma_{\mathrm{aFo}}=\frac{6\mathrm{FL}}{{\mathrm{Wt}}^{2}}$$

The maximum deflection which occurs at the microcantilever tip (*x* = *L*) can be expressed as:

(8) $$z_{\mathrm{aF}\;\max}=\frac{4{\mathrm{FL}}^{3}}{{\mathrm{EWt}}^{3}}$$

For a bending moment *M* exerted on the rectangular microcantilever tip (*x* = *L*), the solution of Equation (1), denoted by *z_aM_*(*x*), subject to boundary conditions given by Equation 3(a,b) is the following:

(9) $$z_{\mathrm{aM}}\;\left(x\right)=\left(\frac{6{\mathrm{ML}}^{2}}{{\mathrm{EWt}}^{3}}\right){\left(\frac{x}{L}\right)}^{2}$$

The surface stress at the base section which is denoted by *σ_aMo_* is equal to:

(10) $$\sigma_{\mathrm{aMo}}=\frac{6M}{{\mathrm{Wt}}^{2}}$$

The maximum deflection which is the deflection at the microcantilever tip is equal to:

(11) $$z_{\mathrm{aM}\;\max}=\frac{6{\mathrm{ML}}^{2}}{{\mathrm{EWt}}^{3}}$$

When the microcantilever is coated on one side with a thin film of receptor, it is usually bent due to analyte adhesion on that layer. This adhesion causes a differential in the surface stress across the microcantilever section yielding a bending moment at each section. The bending moment *M* [2,22] is given by:

(12) $$M=\frac{\Delta \sigma \mathrm{Wt}}{2}$$

where *Δσ* is the difference between the surface stresses of the top and bottom sides of the microcantilever. The solution of Equation (1), denoted by *z_aΔσ_*(*x*), subject to boundary conditions given by Equation 3(a,b) can then be expressed as:

(13) $$z_{a\Delta \sigma }\;\left(x\right)=\frac{6\left(1-\nu \right)\Delta \sigma_{o}L^{2}}{{\mathrm{Et}}^{2}\left(n+1\right)\left(n+2\right)}{\left(\frac{x}{L}\right)}^{n+2}$$

This is because the effective elastic modulus for this case is given by *Y* = *E*/(1−*v*). Also, Δσ is considered to vary along the microcantilever length according to the following relationship:

(14) $$\Delta \sigma ={\Delta \sigma }_{o}{\left(\frac{x}{L}\right)}^{n}$$

where *n* is the model index. This variation is expected as analyte concentration in the surrounding environment is expected to increase as the distance from the microcantilever base increases. The maximum deflection due to analyte adhesion is obtained from Equation (13) by substituting *x = L*. It is equal to:

(15) $$z_{a\Delta \sigma \;\max}\;\left(x\right)=\frac{6\left(1-\nu \right){\Delta \sigma }_{o}L^{2}}{{\mathrm{Et}}^{2}\left(n+1\right)\left(n+2\right)}$$

Equation (15) is reducible to Stoney’s equation when *n* is set to be equal to *zero*.

### 2.2. Microcantilevers with more than One Piece (MC Assemblies)

#### 2.2.1. The Microcantilever Assembly (b)

The geometry of the microcantilever assembly (b) is shown in Figure 1(b). Equation (1) is changeable to the following when the center line of the free end (*x* = *L*) is loaded by a normal concentrated force of magnitude *F*:

(16) $$\frac{d^{2}z_{\mathrm{bF}}}{{\mathrm{dx}}^{2}}=\left(\frac{3\mathrm{FL}}{{\mathrm{EWt}}^{3}}\right)\times 2\left(1-x/L\right)/\cos^{3}\left(\theta \right)$$

Note that *I* for each beam is *I* = *Wt*^3^/12. Note that *θ* is half the triangular tip angle. The cosine of the angle *θ* is given by:

(17) $$\cos\left(\theta \right)={\left[1+0.25{\left[\frac{B/L}{1-0.5\left(W/L\right)}\right]}^{2}\right]}^{-1/2}$$

The boundary conditions for Equation (16) are given by:

(18a,b) $$z_{b}\;\left(x=0\right)={\frac{{\mathrm{dz}}_{b}}{\mathrm{dx}}|}_{x=0}=0$$

The solution of Equation (16), denoted by *z_bF_*(*x*), subject to the above boundary conditions is the following:

(19) $$z_{\mathrm{bF}}\;\left(x\right)=\left(\frac{3{\mathrm{FL}}^{3}}{{\mathrm{EWt}}^{3}}\right)\left[{\left(\frac{x}{L}\right)}^{2}-\frac{1}{3}{\left(\frac{x}{L}\right)}^{3}\right]\left(\frac{1}{\cos^{3}\left(\theta \right)}\right)$$

Using Equation (6), the surface stress at *x* = 0, *σ_bFo_*, is equal to:

(20) $$\sigma_{\mathrm{bFo}}=\left(\frac{3\mathrm{FL}}{{\mathrm{Wt}}^{2}}\right)\left[\frac{1}{\cos\left(\theta \right)}\right]$$

The maximum deflection occurs at the tip (*x* = *L*). It is equal to:

(21) $$z_{\mathrm{bF}\;\max}=\left(\frac{3{\mathrm{FL}}^{3}}{{\mathrm{EWt}}^{3}}\right)\left\{\frac{2}{3\;\cos^{3}\left(\theta \right)}\right\}$$

For a bending moment *M* about *x*-axis exerted on the center line of the free end of the assembly (b) (at *x* = *L*), Equation (1) is changeable to the following form:

(22) $$\frac{d^{2}z_{\mathrm{bM}}}{{\mathrm{dx}}^{2}}=\left(\frac{3M}{{\mathrm{EWt}}^{3}}\right)\times 2/\cos\left(\theta \right)$$

The solution of Equation (22), subject to boundary conditions given by Equation 18(a,b) is the following:

(23) $$z_{\mathrm{bM}}\;\left(x\right)=\left(\frac{3{\mathrm{ML}}^{2}}{{\mathrm{EWt}}^{3}}\right){\left(\frac{x}{L}\right)}^{2}\left[\frac{1}{\cos\left(\theta \right)}\right]$$

As such, the maximum deflection is expected to be equal to:

(24) $$z_{\mathrm{bM}\;\max}=\left(\frac{3{\mathrm{ML}}^{2}}{{\mathrm{EWt}}^{3}}\right)\left\{\frac{1}{\cos\left(\theta \right)}\right\}$$

Using Equation (6), the surface stress at *x* = 0, *σ_cMo_*, is equal to:

(25) $$\sigma_{\mathrm{bMo}}=\frac{3M}{{\mathrm{Wt}}^{2}}\cos\left(\theta \right)$$

When a receptor layer is coated on one side of assembly (b)-side beams (SB), Equation (1) changes to the following form after the analyte adhesion on these coatings:

(26) $$\frac{d^{2}z_{b\Delta \sigma }}{{\mathrm{dx}}^{2}}=\left\{\frac{6\left(1-\nu \right){\Delta \sigma }_{o}}{{\mathrm{Et}}^{2}}\right\}\times {\left(x/L\right)}^{n}/\cos^{2}\left(\theta \right)$$

The solution of Equation (26), subject to boundary conditions given by Equation 18(a,b) is the following:

(27) $$z_{b\Delta \sigma }\;\left(x\right)=\left\{\frac{6\left(1-\nu \right){\Delta \sigma }_{o}L^{2}}{{\mathrm{Et}}^{2}\left(n+1\right)\left(n+2\right)}\right\}{\left(\frac{x}{L}\right)}^{n+2}\left[\frac{1}{\cos^{2}\left(\theta \right)}\right]$$

The maximum deflection due to analyte adhesion is then equal to:

(28) $$z_{b\Delta \sigma \;\max}=\left\{\frac{6\left(1-\nu \right){\Delta \sigma }_{o}L^{2}}{{\mathrm{Et}}^{2}}\right\}\left\{\frac{1/\cos^{2}\left(\theta \right)}{\left(n+1\right)\left(n+2\right)}\right\}$$

Define the first deflection indicator *γ_pU_* as the ratio of the microcantilever deflection at the tip (*x* = *L*) per surface stress at the base for the microcantliever of type (*p*) due to force loading of type *U* to the corresponding value for the rectangular microcantilever. The type (*p*) can be either the microcantilever of type (b) and (c) as shown in Figure 1. The force loading of type *U* can be either concentrated force loading (*F*), external bending moment (*M*) or constant surface stress (*Δσ_o_*). As such, *γ_bF_*, *γ_bM_* and *γ*_*b*Δσ_*o*__ are equal to:

(29a) $$\gamma_{\mathrm{bF}}=1/\cos^{3}\;\left(\theta \right)$$

(29b) $$\gamma_{\mathrm{bM}}=\frac{1}{\cos^{2}\;\left(\theta \right)}$$

(29c) $$\gamma_{{b\Delta \sigma }_{o}}=1/\cos^{2}\;\left(\theta \right)$$

#### 2.2.2. The Microcantilever ɛ-Assembly (Assembly c)

The geometry of the microcantilever assembly (c) is shown in Figure 1(c). Let the centerline of the assembly free end (*x* = *L*) to be loaded by a normal concentrated force of magnitude *F*. And Let the free end of the intermediate beam (IB) be loaded by the negative of the previous load (−*F*). Accordingly, Equation (1) changes to the following:

(30) $$\frac{d^{2}z_{\text{cF}}}{{\text{dx}}^{2}}=\left(\frac{3FL}{{\text{EWt}}^{3}}\right)\times [\begin{array}{c} 2/\text{cos}\left(\theta \right),\;\left(\text{for SB}\right)\\ -4\left(x/L\right),\;\left(\text{for IB}\right) \end{array}$$

where SB stands for the side beams of the assembly. The boundary conditions of Equation (30) are given by:

(31a) $$z_{\mathrm{cSB}}\;\left(x=0\right)={\frac{{\mathrm{dz}}_{\mathrm{cSB}}}{\mathrm{dx}}|}_{x=0}=0$$

(31b) $$z_{\mathrm{cSB}}\;\left(x=L\right)=z_{\mathrm{cIB}}\;\left(x=L\right)$$

(31c) $${\frac{{\mathrm{dz}}_{\mathrm{cSB}}}{\mathrm{dx}}|}_{x=L}={\frac{{\mathrm{dz}}_{\mathrm{cIB}}}{\mathrm{dx}}|}_{x=L}$$

The solution of Equation (30), denoted by *z_cF_* (*x*), is equal to:

(32a) $$z_{\mathrm{cSBF}}\;\left(x\right)=\left(\frac{3{\mathrm{FL}}^{3}}{{\mathrm{EWt}}^{3}}\right){\left(\frac{x}{L}\right)}^{2}\left[\frac{1}{\cos\left(\theta \right)}\right]$$

(32b) $$z_{\mathrm{cIBF}}\;\left(x\right)=\left(\frac{3{\mathrm{FL}}^{3}}{{\mathrm{EWt}}^{3}}\right)\left\{-\left(\frac{2}{3}\right){\left(\frac{x}{L}\right)}^{3}+2\left(\frac{1}{\cos\left(\theta \right)}+1\right)\left(\frac{x}{L}\right)+D_{1}\right\}$$

where *D*_1_ is equal to:

(32c) $$D_{1}=-\left\{\frac{1}{\cos\left(\theta \right)}+\frac{4}{3}\right\}$$

The surface stress at the base section *σ_cFo_* is equal to:

(33) $$\sigma_{\mathrm{cFo}}=\left(\frac{3\mathrm{FL}}{{\mathrm{Wt}}^{2}}\right)\cos\left(\theta \right)$$

Define the second deflection indicator *λ_cU_* as the ratio of the IB-free end deflection *z_cIBU_* (*x* = 0) to that at the assembly free end *z_cU_* (*x* = *L*) due to force loading of type *U*. The force loading of type *U* can be either the current described force loading (*F*), external bending moment loading (*M*) or the constant surface stress (*Δσ*_o_) loading. The last two types of force loadings will be described later on. As such, *λ_cF_* is equal to:

(34) $$\lambda_{\mathrm{cF}}=\frac{{\mathrm{zc}}_{\mathrm{cIBF}}\;\left(x=0\right)}{z_{\mathrm{cF}}\;\left(x=L\right)}=-\left\{1+\frac{4}{3}\cos\left(\theta \right)\right\}$$

Now, let a bending moment *M* be exerted on the assembly (c) free end centerline. And let another bending moment of same magnitude be exerted on the IB-free end at *x* = 0. The deflection equations for this assembly under the current moments loading is given by the following:

(35) $$\frac{d^{2}z_{\text{cM}}}{{\text{dx}}^{2}}=\left(\frac{6M}{{\text{EWt}}^{3}}\right)\times [\begin{array}{c} 2/\text{cos}\left(\theta \right),\;\left(\text{for SB}\right)\\ -2,\;\;\;\left(\text{for IB}\right) \end{array}$$

The boundary conditions are given by Equations 31(a–c). The solution of Equation (35) is given by:

(36a) $$z_{\mathrm{cSBM}}\;\left(x\right)=\frac{1}{\cos\left(\theta \right)}\left(\frac{6{\mathrm{ML}}^{2}}{{\mathrm{EWt}}^{3}}\right){\left(\frac{x}{L}\right)}^{2}$$

(36b) $$z_{\mathrm{cIBM}}\;\left(x\right)=\left(\frac{6{\mathrm{ML}}^{2}}{{\mathrm{EWt}}^{3}}\right)\left\{-{\left(\frac{x}{L}\right)}^{2}+2\left(\frac{1}{\cos\left(\theta \right)}+1\right)\left(\frac{x}{L}\right)+D_{2}\right\}$$

where *D*_2_ is equal to:

(36c) $$D_{2}=-\left[\frac{1}{\cos\left(\theta \right)}+1\right]$$

The surface stress at *x =* 0, *σ_cMo_*, is equal to:

(37) $$\sigma_{\mathrm{cMo}}=\frac{6M}{{\mathrm{Wt}}^{2}}\cos\left(\theta \right)$$

The second deflection indicator for assembly (c) for the current moments loading *λ_cM_* is equal to:

(38) $$\lambda_{\mathrm{cM}}=\frac{z_{\mathrm{cIBM}}\;\left(x=0\right)}{z_{\mathrm{cM}}\;\left(x=L\right)}=-\left[\cos\left(\theta \right)+1\right]$$

If the top surfaces of the side beams of assembly (c) are coated with a receptor while the receptor coating on the intermediate beam is on its bottom surface, then the deflection equations of assembly (c) changes to:

(39) $$\cos^{2}\left(\theta \right)\times \frac{d^{2}z_{\mathrm{cSB}\Delta \sigma }}{{\mathrm{dx}}^{2}}=-\frac{d^{2}z_{\mathrm{cIB}\Delta \sigma }}{{\mathrm{dx}}^{2}}=\frac{6\left(1-\nu \right){\Delta \sigma }_{o}}{{\mathrm{Et}}^{2}}{\left(\frac{x}{L}\right)}^{n}$$

The solution for Equation (39) subject to boundary conditions given by Equation 31(a–c) is equal to:

(40a) $$z_{\mathrm{cSB}\Delta \sigma }\;\left(x\right)=\left\{\frac{6\left(1-\nu \right){\Delta \sigma }_{o}L^{2}}{E\left(n+1\right)t^{2}}\right\}{\left(\frac{x}{L}\right)}^{n+2}\left\{\frac{1/\cos^{2}\left(\theta \right)}{n+2}\right\}$$

(40b) $$z_{\mathrm{cIB}\Delta \sigma }\;\left(x\right)=\left\{\frac{6\left(1-\nu \right){\Delta \sigma }_{o}L^{2}}{E\left(n+1\right)t^{2}}\right\}\left\{\frac{-1}{\left(n+2\right)}{\left(\frac{x}{L}\right)}^{n+2}+\left[1+\frac{1}{\cos^{2}\left(\theta \right)}\right]\left[\frac{x}{L}-\frac{n+1}{n+2}\right]\right\}$$

The deflection indicator for assembly (c) due to the alternating analyte adhesion on the surfaces *λ_cΔσ_* is equal to:

(41) $$\lambda_{c\Delta \sigma }=\frac{z_{\text{cIB}\Delta \sigma }\;\left(x=0\right)}{z_{\text{cSB}\Delta \sigma }\;\left(x=L\right)}=-\left(n+1\right)\left[{\text{cos}}^{2}\left(\theta \right)+1\right]$$

The deflection indicators *γ_cF_*, *γ_cM_* and *γ*_*cΔσ*_*o*__ can be shown to be equal to the following:

(42a) $$\gamma_{\mathrm{cF}}=1.5/\cos^{2}\left(\theta \right)$$

(42b) $$\gamma_{\mathrm{cM}}=1/\cos^{2}\left(\theta \right)$$

(42c) $$\gamma_{{c\Delta \sigma }_{o}}=1/\cos^{2}\left(\theta \right)$$

## 3. Results and Discussion

### 3.1. Validation of the Results

**Figure 2.**
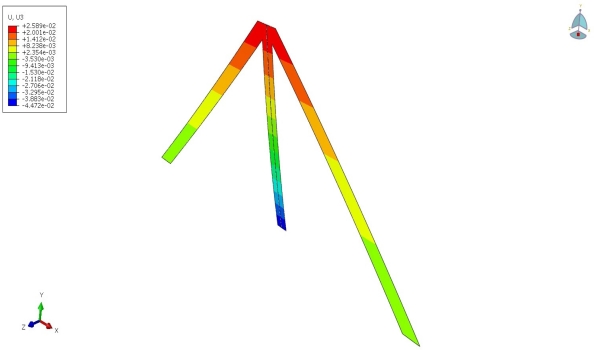
Deflection profile for assembly (c) using numerical solutions with *L =* 385 μm, *W* = 30 μm, *t* = 20 nm, *M* = 10^−12^ Nμm, *E* = 0.185 Nμm^−2^ and ν = 0.33, deflections (U) are in μm.

The present analytical methods were tested against an accurate numerical solution using finite element methods and accounting for all mechanical constraints induced by the assemblies. Among these constraints is restraining the wrapping of the side beams due to the presence of the small connecting beam at *x* = *L*. The deflection contours for assembly (c) with *L* = 385 μm, *W* = 30 μm and *t* = 20 nm under concentrated moment condition described in section 2.2.2 with *M* = 10^−12^ Nμm is shown in Figure 2. The microcantilever material was taken to be silicon with *E* = 0.185 Nμm^−2^ and a poisons ratio of *ν* = 0.33. The assembly deflection at *x = L* is equal to *z_cM_* (*x* = *L*) = 0.028 μm using Equation (36b). Also, the deflection at the intermediate beam’s free end can be shown to be equal to *z_cIBM_* (*x* = *0*) = 0.048 μm. As can be seen from Figure 2, the corresponding numerical values of those deflections are equal to 0.026 μm and 0.045 μm, respectively. Notice that the maximum error between the numerical and the derived analytical solutions is less than 10 percent. Also, notice that the numerical values of deflections are smaller than those predicated by the analytical methods. This is because the geometrical constraints imposed on the assemblies impede the deflections. (Legand)

### 3.2. Discussion of the Results

#### 3.2.1. Discussion of the Results of First Performance Indicators

**Figure 3.**
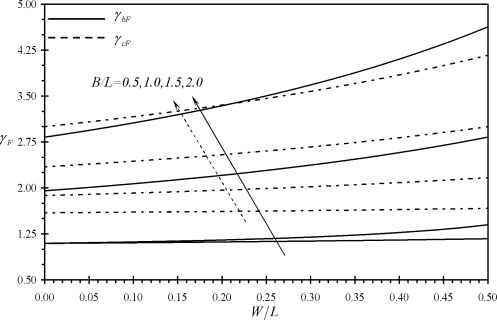
Effects of the relative dimensions of the microcantilevers assemblies (b) and (c) on the first performance indicators *γ_bF_* and *γ_cF_*.

**Figure 4.**
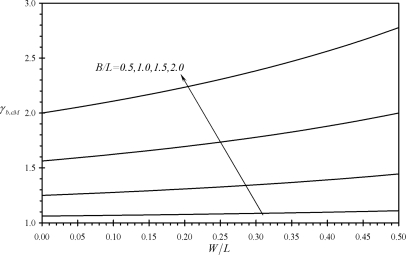
Effects of the relative dimensions of the microcantilevers assemblies (b) and (c) on the first performance indicators *γ_bM_* and *γ_cM_*.

**Figure 5.**
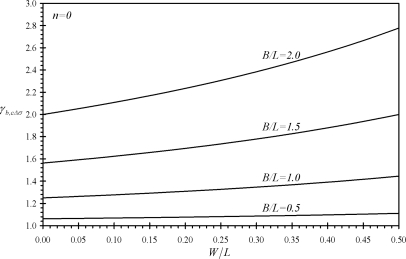
Effects of the relative dimensions of the microcantilevers assemblies (b) and (c) on the first performance indicators *γ_bΔσ_* and *γ_cΔσ_*.

Figure 3 shows the variation of the performance indicators *γ_bF_* and *γ_cF_* with the relative dimensions of assemblies (b) and (c). It is noticed that all the values of *γ_bF_* and *γ_cF_* are larger than one which indicates that assemblies (b) and (c) produce larger deflections than rectangular microcantilevers under same surface stress at the base and same extension length *L*. Moreover, both indicators increase as both the microcantilever width *W* and the assembly width *B* increase. Similar findings are noticed for the performance indicators *γ_bM_*, *γ_cM_*, *γ_bΔσ_* and *γ_cΔσ_* as can be seen from Figures 4 and 5. On the other hand, an increase in *B* causes the effective free length of the assembly to increase, which makes the assembly more sensitive to external noises.

#### 3.2.2. Discussion of the Results of Second Performance Indicators

**Figure 6.**
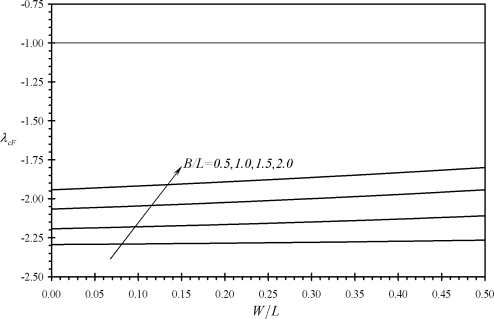
Effects of the relative dimensions of the microcantilevers assemblies (b) and (c) on the second performance indicators *λ_cF_*.

**Figure 7.**
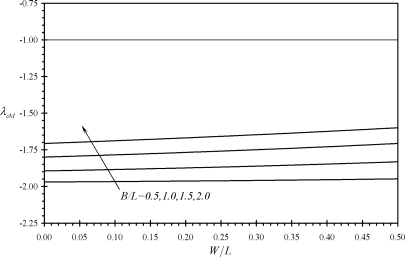
Effects of the relative dimensions of the microcantilevers assemblies (b) and (c) on the second performance indicators *λ_cM_*.

**Figure 8.**
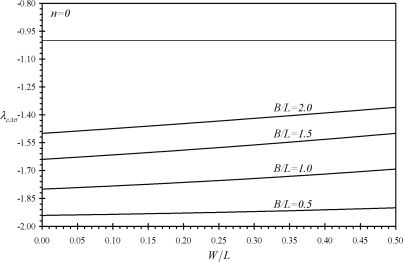
Effects of the relative dimensions of the microcantilevers assemblies (b) and (c) on the second performance indicators *λ_cΔσ_*.

Figure 6 shows the variation of the second performance indicator *λ_cF_* with the relative dimensions of assembly (c). It is noticed that all values of *λ_cF_* are smaller than minus one. This indicates that IB-free end deflection is always larger than that of the assembly tip deflection. Moreover, the absolute value of *λ_cF_* is noticed to increases as both *W* and *B* decreases. Similar findings are noticed for the performance indicators *λ_cM_* and *λ_cΔσ_* as can be seen from Figures 7 and 8. As a result, assembly (c) can provide larger deflections than assembly (b) while it is less affected by external noise. This is because its deflection increase as *B* decreases which results in a reduction of the assembly’s free length. Moreover, the absolute values of *λ_cΔσ_* increases as *n* increases as can be shown using Equation (41). This indicates the advantage of assembly (c) in microsensing applications as compared to rectangular cantilevers or triangular cantilevers.

## 4. Conclusions

A theoretical investigation on improving deflections of microcantilevers sensors is presented in this work based on analytical solutions. Three different mcirocantilevers were analyzed. These are: (a) the rectangular microcantilever, (b) the modified triangular microcantilever assembly, and (c) the ɛ-microcantilever assembly. The deflection theory of thin beams is utilized to obtain the deflection profile for each microcantilever. Different force loadings were considered including concentrated force, concentrated moment and constant surface stress. Different deflection indicators were defined and computed. It was found that both the modified triangular microcantilever assembly and the ɛ-microcantilever assembly produce larger deflections than the rectangular microcantilever under the same base surface stress and same extension length. The deflection of the former microcantilevers can be 280% and 425% above that of the rectangular microcantilever for concentrated moment and constant surface stress cases, respectively. In addition, the ɛ-microcantilever assembly was found to produce larger deflection than the triangular microcantilever assembly. The deflection of the ɛ-microcantilever intermediate free end may reach 200% above that of the triangular microcantilever assembly. It was found that deflection enhancement due to ɛ-microcantilever increases as the assembly free length decreases. The cited conclusions were found to be valid for the different force loading conditions. The analytical results were validated against an accurate numerical solution utilizing a finite element method. The analytical and numerical solutions were found to be in good agreement. Based on our analysis, the ɛ-microcantilever assembly was found to provide a superior and the best favorable high detection capability with the least susceptibility to external noise in microsensing applications. As such, it is recommended to experimentally test it to show whether detective potential of microsensors can be increased.

## Nomenclature

*B*: Base length of the microcantilever assembly
*E*: Elastic modulus (N μm^−2^)
*F*: concentrated force (N)
*I*: Area moment of inertia (μm^4^)
*L*: microcantilever or assembly extension length (μm)
*M*: moment (N μm)
*n*: surface stress model index
*t*: microcantilever thickness (μm)
*W*: microcantilever width (μm)
*x*: axis of the extension dimension (μm)
*Y*: effective elastic modulus (N μm^−2^)
*z*: deflection (μm)

**Greek Symbols**
*γ*: first deflection indicator
*λ*: second deflection indicator
*ν*: Poisson’s ratio
*σ*: surface stress

**Subscripts**
*F*: concentrated force condition
*M*: moment condition
*Δσ*: constant differential surface stress condition

**Abbreviations**
*IB*: the intermediate beam of assembly (c)
*SB*: the side beams of assembly (c)
